# Individuals with Wiedemann-Steiner syndrome show nonverbal reasoning and visuospatial defects with relative verbal skill sparing

**DOI:** 10.1017/S1355617722000467

**Published:** 2022-09-05

**Authors:** Rowena Ng, Jacqueline Harris, Jill A. Fahrner, Hans Tomas Bjornsson

**Affiliations:** 1 Kennedy Krieger Institute, Baltimore, MD, USA; 2 Department of Psychiatry and Behavioral Sciences, Johns Hopkins University School of Medicine, Baltimore, MD, USA; 3 Department of Neurology, Johns Hopkins University School of Medicine, Baltimore, MD, USA; 4 Department of Genetic Medicine, Johns Hopkins University School of Medicine, Baltimore, MD, USA; 5 Department of Pediatrics, Johns Hopkins University School of Medicine, Baltimore, MD, USA; 6 Faculty of Medicine, University of Iceland, Reykjavik, Iceland; 7 Landspitali University Hospital, Reykjavik, Iceland

**Keywords:** Genetics, Genetic disorders, Clinical neuropsychology, Hippocampus, Visuospatial functioning, Executive functioning

## Abstract

**Objectives:**

Wiedemann-Steiner syndrome (WSS) is a rare Mendelian disorder of the epigenetic machinery caused by heterozygous pathogenic variants in *KMT2A*. Currently, the specific neurocognitive profile of this syndrome remains unknown. This case series provides insight into the cognitive phenotype of WSS.

**Methods:**

This study involves a retrospective medical chart review of 10 pediatric patients, each with a molecularly confirmed diagnosis of WSS who underwent clinical neuropsychological evaluation at an academic medical center.

**Results:**

The majority of patients performed in the below average to very low ranges in Nonverbal Reasoning, Visual/Spatial Perception, Visuoconstruction, Visual Memory, Attention, Working Memory and Math Computation skills. In contrast, over half the sample performed within normal limits on Receptive Vocabulary, Verbal Memory, and Word Reading. Wilcoxon signed rank test showed weaker Nonverbal versus Verbal Reasoning skills (*p* = .005). Most caregivers reported deficits in executive functioning, most notably in emotion regulation.

**Conclusions:**

Nonverbal reasoning/memory, visuospatial/construction, attention, working memory, executive functioning, and math computation skills are areas of weakness among those with WSS. These findings overlap with research on Kabuki syndrome, which is caused by variants in *KMT2D,* and suggest disruption in the neurogenesis of the hippocampal formation may drive shared pathogenesis of the two syndromes.

## Introduction

Wiedemann-Steiner syndrome (WSS, MIM #605130) is a Mendelian Disorder of Epigenetic Machinery (MDEM) most often caused by haploinsufficiency of *KMT2A*, a gene encoding a histone methyltransferase that adds histone 3 lysine 4 (H3K4) methylation (Jones et al., 2012). In turn, disruption in chromatin remodeling results in secondary wide-spread abnormal gene expression and multisystemic developmental effects. This autosomal dominant disorder largely occurs de novo and is thought to affect males and females equally (Jones et al., 2012).

WSS is extremely rare, which contributes to the challenges in characterizing the neurocognitive phenotype associated with this syndrome. While the first case of WSS was described in 1989 (Wiedemann et al., 1989), it was not until 2012 that the genetic basis was discovered (Jones et al., 2012). Hallmark characteristics of WSS includes hypertrichosis (arms, elbows and back), distinctive facial features (thick eyebrows, narrow palpebral fissures, wide spaced eyes, long eyelashes), and postnatal growth deficiency (Baer et al., 2018; Jones et al., 2012; Miyake et al., 2016). Most notably, in a recent review of the largest clinical sample of patients with WSS to date, developmental delay and/or intellectual disability (mild to severe) was the most frequently observed feature among affected individuals, occurring in 97% of the clinical sample (Sheppard et al., 2021). However, to date, the specific neurocognitive profile associated with WSS remains elusive, as published literature largely involved retrospective review of medical charts (i.e., diagnoses made by a clinician) or natural history data.

Characterizing the neurocognitive and behavioral phenotypes associated with WSS is an important step towards elucidating the pathogenesis of cognitive disorders. Specifically, identifying particular cognitive functions impacted in this syndrome can offer clues on the downstream effect the disrupted epigenetic machinery has on neurodevelopment. For example, recent research involving Kabuki syndrome (KS), a disease caused by pathogenic variants in *KMT2D,* a gene with similar structure and function as *KMT2A*, was found to be associated with visuospatial deficits, suggestive of an aberration in the neurogenesis of the dentate gyrus (Harris et al., 2019), which was consistent with memory impairment and histopathologic anomalies of the hippocampus in mouse models of this syndrome (Bjornsson et al., 2014). In addition, the cognitive dysfunction and hippocampal neurogenesis deficits in mouse models of KS has been reported to be postnatally correctible with administration of a histone deacetylase inhibitor (Bjornsson et al., 2014) or lysine-specific demethylase 1A inhibitor (Zhang et al., 2021), and adopting a ketogenic diet (Benjamin et al., 2017). Consequently, cognitive research involving individuals with KS and other disorders, such as that of Harris and colleagues (2019), is critical to inform therapeutic targets, further refining research done in animal models of the disease. Collectively, these findings in KS suggest that cognitive dysfunction associated with other MDEMs such as WSS may be reversible with appropriate treatment. Therefore, ascertaining the specific neuroanatomical and functional deficiencies linked to WSS will be central to the development of targeted clinical trials.

To this end, this case series aims to offer a more detailed description of the cognitive phenotype associated with WSS. Specifically, this investigation involves a retrospective chart review of neuropsychological assessment data in a clinical sample of patients with molecular confirmation of the genetic disorder. There is a suspicion of greater deficits in nonverbal and visuospatial skills, akin to the cognitive phenotype of KS (Harris et al., 2019). This is particularly interesting given the structural similarities of the disease-relevant *KMT2D* (KS) and *KMT2A* (WSS) proteins involved in H3K4 histone methylation, which has a regulatory role in hippocampal functions in rodent models (Gupta et al., 2010). Indeed, knockdown of *KMT2A* in mice similarly yielded memory impairment consistent with hippocampal dysfunction (Kerimoglu et al., 2017). Additionally, neuronal deletion of *KMT2A* in the prefrontal cortex, ventral striatum, and nucleus accumbens has been associated with working memory deficits and increased anxiety in mouse models, implicating that normal neurogenesis may rely on the downregulation of *KMT2A* (Jakovcevski et al., 2015; Shen et al., 2016). Accordingly, drawing from limited research with animal models as well as anecdotal experience in patients, we also anticipate deficits in executive functioning and emotion regulation.

## Methods

### Clinical sample

This case series involved a retrospective chart review of 10 pediatric patients (4F; *M*_age at testing_ = 10.81 years, *SD* = 3.49; range: 6 to 17) with a molecularly confirmed diagnosis of WSS who were evaluated by the Department of Neuropsychology at Kennedy Krieger Institute (KKI) and followed by the Neurogenetics Clinic at KKI and/or the Epigenetics and Chromatin Clinic at Johns Hopkins University School of Medicine (JHUSOM). The racial composition of our sample was largely White (White, *N* = 9; Black, *N* = 1). All molecular testing was reviewed by a KKI or JHUSOM physician to confirm pathogenicity (whole exome sequencing, *N* = 9; single gene sequencing, *N* = 1) and all patients had at least one physical exam by those physicians to confirm the diagnosis of WSS. Majority of the patients had a pathogenic variant (*N* = 9), and one had a variant classified by the reporting laboratory as being of uncertain significance. However, we are confident that the latter variant is likely pathogenic and causing WSS in this individual because the patient’s findings are consistent with a diagnosis of WSS; it occurred *de novo* in the patient (i.e., it is not present in the unaffected parents); it has not been reported in large population databases like Genome Aggregation database (gnomAD), which report benign genetic variation; and a different amino acid substitution at the same position has been reported as pathogenic in an individual with features of WSS (Baer et al., 2018). Data of those with additional genetic anomalies such as microdeletions or chromosomal duplications were excluded to ensure the observed neurobehavioral patterns are specific to WSS. Our clinical sample included largely individuals with *de novo* variants in *KMT2A* (*N* = 7), one inherited from a parent with proven mosaicism, and two unknown inheritance. For more information on gene variant and pathogenicity stage, please see Supplementary Table 1.

Based on a review of the original assessment reports, all patients presented with functional speech at the time of the appointment. Additionally, vision and hearing were determined adequate for testing by clinicians. At the time, patient’s caregivers reported no significant concerns with vision or hearing. All patients were determined to show adequate effort, and test results were considered valid estimations of cognitive functioning based on the original clinician’s judgment. Out of our sample, eight patients had a diagnosis of intellectual disability and three with autism spectrum disorder.

This study was approved by the Institutional Review Board at JHU, and is in accordance with the Helsinki Declaration. Informed consent and/or assent were obtained by patient’s legal guardian and/or the patient prior to inclusion in the study.

### Procedure and materials

All patients completed a comprehensive neuropsychological evaluation with a licensed psychologist at KKI as part of their clinical care. In total, four neuropsychologists completed testing with our patient sample and each provided a battery of cognitive measures based on their clinical judgment. Largely, as shown in Table 1, most patients completed a measure of intellectual functioning yielding nonverbal and verbal reasoning indexes, receptive vocabulary, visual/spatial perception, visuoconstruction, verbal learning/memory, visual memory, working memory, attention, and select academic skills (sight word reading, math calculation skills). Caregivers also completed a standardized parent-rating inventory to assess day-to-day executive functioning and adaptive skills.

Table 1 outlines the proportion of patients that completed each cognitive domain and tests administered. Half of the patients were evaluated via home-based teleneuropsychological testing with HIPAA-compliant videoconferencing platform (Zoom) during the COVID-19 pandemic. Those evaluated via telehealth modality were given a comparable test battery administered over one to two sessions, whereas patients seen face-to-face completed testing in a single session. The individuals who completed a teleneuropsychological assessment were administered subtests from the Wechsler Intelligence Scale for Children 5^th^ Edition (WISC-V) and WISC-V Integrated to yield a WISC-V Verbal Comprehension Index and WISC-V Nonmotor Nonverbal Index with age-corrected norms (Raiford, 2017).

Supplementary table 2 lists the intellectual functioning composite for 8 of the 10 patients. This estimate was not obtained for two patients as one completed only verbal and nonverbal cluster subtests from the Differential Ability Scales 2^nd^ Edition (DAS-II) School-Age battery and the other completed the out-of-level DAS-II Early Years battery. Both assessments did not yield the DAS-II General Conceptual Ability composite, an index of intellectual functioning. Of the eight patients, four completed WISC-V and WISC-V Integrated subtests through telehealth modality which yielded the Nonmotor General Ability Index (Nonmotor GAI)(Raiford, 2017); two completed WISC-V subtests which were used to generate the GAI, one received an intelligence index through completion of the Reynolds Intellectual Assessment Scales 2^nd^ Edition (RIAS-2), and one had the General Conceptual Ability composite through the DAS-II.

### Data strategy

Across standardized cognitive and academic test measures, the derived performance scores were converted to standard scores and percentile ranks utilizing age-corrected norms in the respective technical and scoring manuals. Given our small sample, non-parametric testing was used. Wilcoxon signed-rank test was applied to determine if a within-group difference in verbal and nonverbal indexes was observed. Finally, ratings provided for the Behavior Rating Inventory of Executive Function (BRIEF) and Behavior Rating Inventory of Executive Function 2^nd^ Edition (BRIEF-2) were converted based on gender- and age-based norms. Subsequently, patients’ objective test performance across each cognitive domain was qualitatively coded based on the resulting percentile rank: *Low to Very Low* for ≤ 2^nd^ percentile, *Below Average* for performance between 3^rd^ and 15^th^ percentile, *Within Normal Limits or Above* when ≥16^th^ percentile. Likewise, resulting standard scores on caregiver inventories were coded for clinical significance or marked functional impairment. On the BRIEF/BRIEF-2, in accordance to the inventory manual, *Clinically Significant* refers to obtained *T*-scores ≥70, *At-Risk* (i.e., Elevated to Potentially Clinically Significant) refers to *T*-scores 60 to 69, and *Within Normal Limits* reflects *T*-scores ≤59.

## Results

### Cognitive test performance

As shown in Table 2, on average, patients with WSS yield stronger performance on verbal/language measures than nonverbal and visual processing tests. Average percentile rank for Receptive Vocabulary and Verbal Memory were within normal limits or above; whereas, mean performance on Nonverbal Index (nonverbal reasoning), Visual and Spatial Perception, Visuoconstruction, and Visual Memory were well below average. Verbal reasoning was slightly below average. Wilcoxon signed-rank test revealed Nonverbal Index scores were significantly lower than Verbal Index scores, *Z* = −2.89, *p* = .005. However, verbal and language measures also yielded more variable performance as reflected by wider range in percentile ranks, as compared to nonverbal and visual perceptual tests. On average, Attention and Working Memory were below average, although the latter also yielded a wide range of outcome scores. Among academic measures, patients with WSS showed relatively stronger sight word reading with performance scores that fell between below average to average, as compared to math computation skills, which was very low to below average.

Supplementary table 2 outlines individual patient’s percentile rank across cognitive domains and their intellectual functioning composite (standard score). As illustrated in the table, the pattern of stronger verbal over nonverbal reasoning skills was observed regardless of testing modality. Specifically, four of the five patients who completed telehealth testing and three of five patients who completed face-to-face or an in-person neuropsychological evaluation showed stronger Verbal than Nonverbal Index scores.

**Figure 1.**
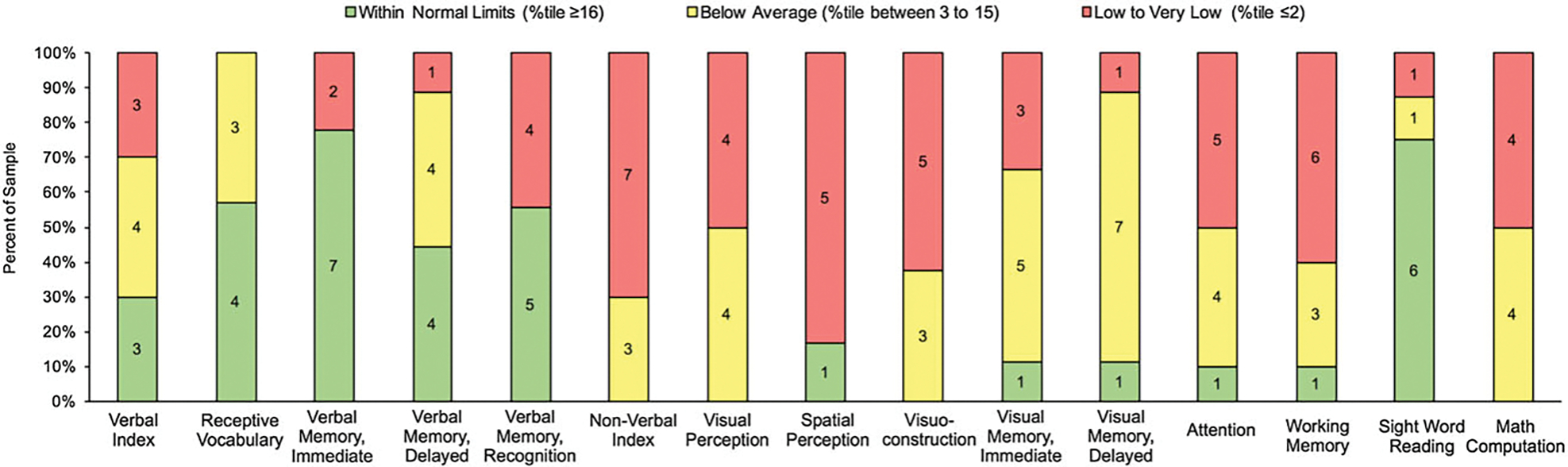
Number of patients performing within normal limits or above, below average, and low to very low across cognitive domains.

Figure 1 reflects the proportion of patients who performed within normal limits or above across cognitive domains. In brief, approximately 30% patients performed within normal limits or above in Verbal Index (3 out of 10), 57.14% in Receptive Vocabulary (4 out of 7), 77.77% in Verbal Immediate Memory Recall (7 out of 9), 44.44% in Verbal Delayed Memory Recall (4 out of 9), 55.55% in Verbal Delayed Recognition (5 out of 9), and 75% in Sight Word Reading (6 out of 8). One patient or 10% of the clinical sample performed within normal limits in Attention and Working Memory, 11.11% in Visual Immediate and Delayed Memory (1 out of 9), and 16.67% in Spatial Perception (1 out of 6). In contrast, no patients performed within broad average or above in Nonverbal Index, Visual Perception, Visuoconstruction, or Math Computation.

### Parent report inventory of executive functioning

**Figure 2.**
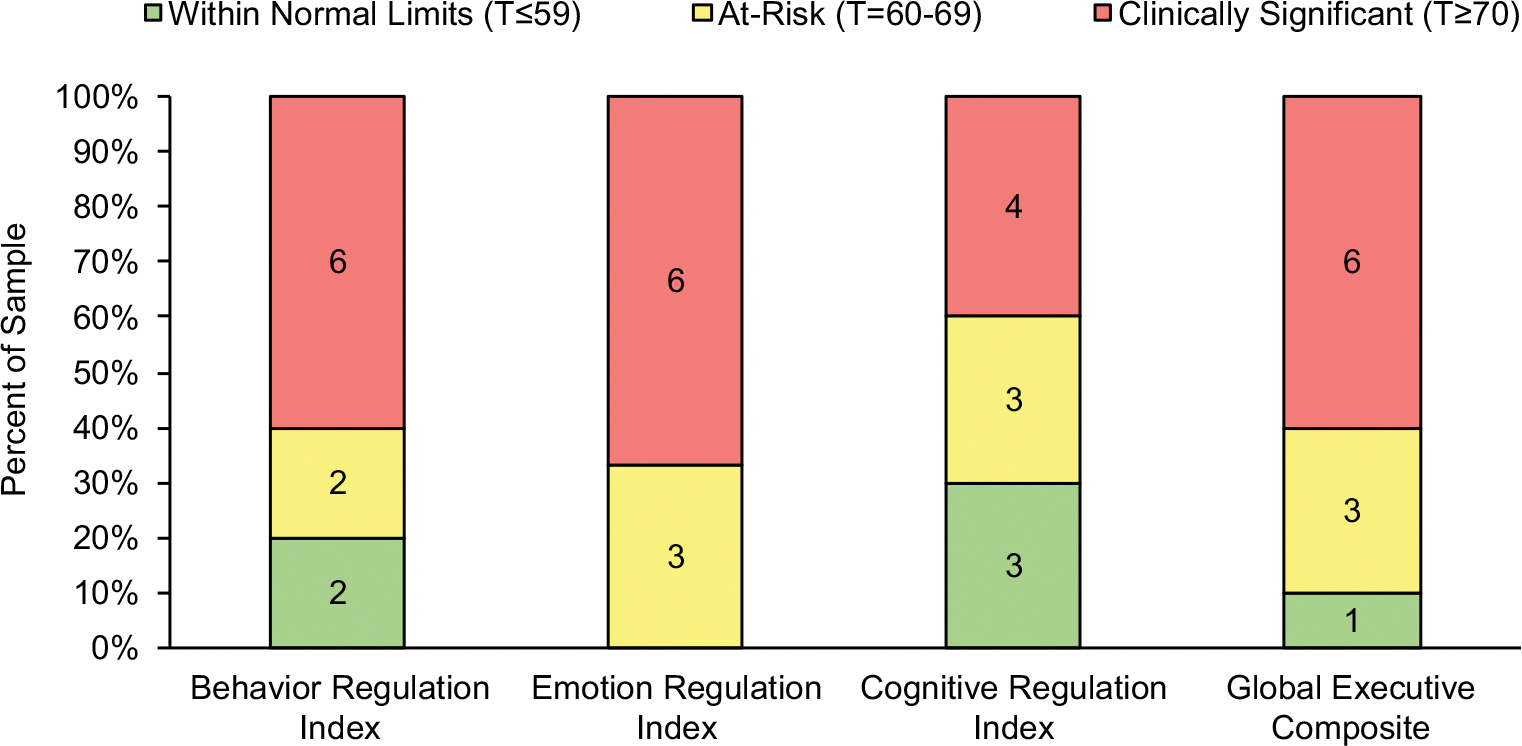
Parent ratings on the Behavior Rating Inventory of Executive Functioning (BRIEF or BRIEF-2).

Figure 2 illustrates the percentage of the clinical sample whose parents’ ratings revealed at-risk to clinically significant problems across executive functioning domains. Of note, one patient completed the BRIEF, which does not yield an Emotion Regulation Index. Overall, the majority of the clinical sample (90%) were rated in the at-risk or clinically significant range for global problems with executive functioning. Across executive domains, most concerns were reported in Emotion Regulation (100% or 9 out of 9), followed by Behavior Regulation (80% or 8 out of 10) and Cognitive Regulation (70% or 7 out of 10). Of note, Pearson bivariate correlations did not reveal associations between intellectual functioning composite and executive domains or the overall BRIEF General Executive Composite.

## Discussion

This case series is the first study to provide a broad overview of the neurocognitive profile associated with pediatric patients with confirmed pathogenic variants in *KMT2A*. In brief, on average, those with WSS showed relatively stronger verbal reasoning, verbal memory and receptive language than nonverbal reasoning, visual memory, and visual-spatial processing. Sight word reading was also observed as an area of relative strength, whereas math computation was a consistent area of academic difficulty. The majority of patients showed below average to very low performances across attention and working memory; and were rated in the at-risk to clinically significant range in day-to-day executive functioning deficits.

Collectively, our clinical sample of pediatric patients with WSS revealed more consistent pattern of impairment in nonverbal reasoning, visual/spatial perceptual, and object learning/memory. While verbal and select language skills appear relatively more intact, greater heterogeneity was observed among these functions. Broadly, our findings may suggest hippocampal dysfunction, particularly given observed deficiencies in visual memory, visuospatial perception and visual integration in both motor and mental construction tasks (Burgess, 2008; Lee et al., 2012; Wicking et al., 2014). These observed cognitive trends are congruent with the phenotypic features of individuals with KS (Harris et al., 2019). As highlighted by Harris and colleagues (2019), these pattern of findings may implicate the effect of the gene mutation on hippocampal, and specifically dentate gyrus, neurogenesis following disruption in methylation of H3K4 and subsequent chromatin remodeling. Notably, other studies involving animal models have observed deficits in hippocampal functions, including memory formation, with deficiency in H3K4 histone methyltransferase (Gupta et al., 2010). However, given the limited cognitive constructs assessed and the mixed tests administered, additional research with larger sample sizes and comparison groups, supplemental metrics of neural functioning (e.g., electroencephalogram, EEG; positron emission tomography, PET), and a comprehensive battery of measures directed towards the examination of hippocampal functions are necessary to ascertain if our observed pattern of cognitive functioning reflects hippocampal dysfunction uniquely or byproducts of other neurodevelopmental anomalies. While neurocognitive testing offers critical clues on brain function that may not be reflected on structural or anatomical imaging studies (e.g., MRI), cognitive functions generally involve multiple neural substrates/circuits. As such, neuropsychological assessment should not be utilized alone to isolate functional deficits to a single brain region. Integration of EEG, PET or fMRI studies with cognitive and behavioral testing will be imperative in subsequent investigations to identify focal brain dysfunction associated with WSS.

Importantly, future cross-syndrome comparisons of the cognitive and behavioral phenotypes associated with mutations in the *KMT2* family of genes (e.g., KS with pathogenic variant of *KMT2D*, intellectual disability or autism related to *KMT2C* mutation)(Vallianatos & Iwase, 2015) and Mendelian Disorders of the Epigenetic Machinery broadly (Fahrner & Bjornsson, 2019; Mossink et al., 2021) will better elucidate the shared pathogenic mechanism that results in ontogenesis of developmental disorders and psychopathology, and likewise, unique disease characteristics. These efforts will better inform targets of syndrome-specific clinical trials, and likewise epigenetic therapies that can be generalized across syndromes with common disease-causing mechanisms.

Impaired working memory and executive functioning based on objective and parent-report measures may also implicate the role of *KMT2A* in the neurogenesis of the prefrontal cortex (Jakovcevski et al., 2015). Executive dysfunction, including poor emotion and behavior regulation, may explain the relatively high rate of hyperactivity (44.3%), and aggressive behaviors (33%) that has recently been documented with a large cohort of patients with WSS (Sheppard et al., 2021), in addition to case reports of autism-like features including rigid and externalizing behaviors but intact social motivation (Chan et al., 2019). Expanding the clinical phenotypic spectrum of WSS by utilizing a developmental approach will be a vital step to discern developmental psychopathology that emerges as downstream effects of the disrupted epigenetic machineries. Moreover, longitudinal approaches in phenotyping efforts are necessary to determine the stability of the neurobehavioral profile, and/or sensitive periods when clinical and behavior interventions may offer more protective effects on long-term cognitive sequelae.

There are limitations in our case series that prospective research should consider. Given the rarity of WSS, our clinical sample is small. A larger sample will be necessary to illustrate precise epigenotype-phenotype associations. Given neuropsychological evaluations were administered to patients for clinical referrals, heterogeneous tests with different task instructions were combined to denote a cognitive domain (e.g., NEPSY-II Arrows offers increasing number of possible response options in later items, whereas JLO has a consistent number of choices). In addition, the assessment batteries provided included limited measures of hippocampal functioning. Although neurocognitive measures are sensitive to functionality of neural substrates (Wicking et al., 2014), these often index a combination of cognitive skills and thus multiple neural systems involved. Thus, future studies should consider providing a targeted battery of tests that assess spatial cognition and memory in addition to episodic memory – all of which are dedicated functions of the hippocampus. Offering a wider range of cognitive assays that collectively implicate various aspects of the hippocampal formation will afford more precise measurements and strengthen the gene-brain-cognitive associations driven by the epigenetic imbalance resulting from *KMT2A* mutation. The heterogeneity of our clinical sample (i.e., wide age range of patients) should also be considered, as cognitive and academic constructs may develop at different pace across select developmental periods. Notably, impairment in select cognitive functions may be associated with differential impact on day-to-day functioning based on the developmental stage. Finally, our clinical sample included a combination of patients evaluated through telehealth and face-to-face modalities. The proportion of those with impairment under the telehealth versus face-to-face in-clinic modality were roughly similar across Verbal Index (2/5 vs. 1/5) and Nonverbal Index (3/5 vs. 4/5). While there are limitations to teleneuropsychology (e.g., there are more limited measures adapted for remote virtual administration than traditional face-to-face testing), the swift adoption of this model of care secondary to the pandemic has revealed its utility in increasing access to clinical services and research for some families affected by rare diseases such as WSS. Nevertheless, more research is needed to support the validity of in-home teleneuropsychological testing particularly among pediatric samples with greater cognitive and physical/sensory impairment, which are typically more prevalent in syndromic intellectual disability.

In brief, our case series with a sample of pediatric patients with WSS observed a distinctive trend of discrepant impairment in nonverbal reasoning, visual-spatial perception, visual memory and math calculation skills that warrant more investigation. Systematic and longitudinal approach to cognitive phenotyping will be necessary to identify if these deficits vary as a function of variant or other molecular characteristics.

## Supplementary Material

Supplement

**Supplementary material.** To view supplementary material for this article, please visit https://doi.org/10.1017/S1355617722000467

## Figures and Tables

**Table 1.** Proportion of patients (*N* = 10) that completed a test measure across cognitive domains

Cognitive domain	Patients that completed a measure	Tests applied

Verbal Reasoning	10	• WISC-V Verbal Comprehension Index (*N* = 6; Wechsler, 2014) • RIAS-2 Verbal Intelligence (*N* = 1; Reynolds & Hamphaus, 2015) • DAS-II Verbal Cluster (*N* = 3; Elliot, 1990)
Nonverbal Reasoning	10	• WISC-V Nonmotor Nonverbal Index (*N* = 4, Wechsler & Kaplan, 2015) • WISC-V Nonverbal Index (*N* = 2; Wechsler, 2014) • DAS-II Nonverbal Cluster (*N* = 3; Elliot, 1990) • RIAS-2 Nonverbal Intelligence (*N* = 1; Reynolds & Hamphaus, 2015)
Receptive Vocabulary Verbal Learning/Memory	7	• PPVT-5 (Dunn, 2018) • ChAMP List (*N* = 8; Sherman & Brooks, 2015)
Immediate/Delayed Recall and Recognition	9	• WRAML-2 Verbal Learning/Delay (*N* = 1, Sheslow & Adams, 2003)
Visual Perception	8	• Beery VMI-6 Visual Perception (Beery, 2004)
Spatial Perception	6	• Benton's Judgment of Line Orientation (*N* = 4; Benton et al., 1978) • NEPSY-II Arrows (*N* = 2; Brooks et al., 2009)
Visuoconstruction	8	• WISC-V Integrated Block Design (*N* = 4, Wechsler & Kaplan, 2015) • WISC-V Block Design (*N* = 2; Wechsler, 2014) • DAS-II Pattern Construction (*N* = 2; Elliot, 1990)
Visual Learning/Memory		
Immediate Recall/Recognition	9	• ChAMP Objects (*N* = 7; Sherman & Brooks, 2015)
Delayed Recall/Recognition	9	• DAS-II Recall of Objects (*N* = 2; Elliot, 1990)
Attention	10	• WISC-V Digit Span Forward (*N* = 6; Wechsler, 2014) • DAS-II Recall of Digits Forward (*N* = 4; Elliot, 1990)
Working Memory	10	• WISC-V Digit Span Backward (*N* = 6; Wechsler, 2014) • DAS-II Recall of Digits Backward (*N* = 4; Elliot, 1990)
Academic Skills		
Sight Word Reading	8	• KTEA-3 Letter & Word Recognition, Math Computation (*N* = 7; Kaufman & Kaufman, 2014)
Math Computation	8	• WIAT-III Word Reading, Numerical Operations (*N* = 1; Wechsler, 2009)
Executive Functioning (Caregiver Report)	10	• BRIEF-2 (*N* = 9; Gioia et al., 2000) • BRIEF (*N* = 1; Gioia et al., 2015)

Abbreviations. Beery VMI-6 = Beery-Buktenica Developmental Test of Visual-Motor Integration 6^th^ Edition, BRIEF = Behavior Rating Inventory of Executive Function, ChAMP = Child and Adolescent Memory Performance, DAS-II = Differential Ability Scales 2^nd^ Edition, KTEA-3 = Kaufman Test of Educational Achievement 3^rd^ Edition, NEPSY-II = A Developmental Neuropsychological Assessment, PPVT-5 = Peabody Picture Vocabulary Test 5^th^ Edition, RIAS-2 = Reynolds Intellectual Assessment Scales 2^nd^ Edition, WISC-V = Wechsler Intelligence Scale for Children 5^th^ Edition, WIAT-III = Wechsler Individual Achievement Test 3^rd^ Edition, WRAML-2 = Wide Range Assessment of Memory and Learning 2^nd^ Edition.

**Table 2.** Average percentile rank across cognitive domain

Cognitive domain	*M*_Percentile Rank_	Standard deviation	Range

Verbal Reasoning	15.40	19.87	1 to 58
Nonverbal Reasoning	2.95	4.34	<1 to 13
Receptive Vocabulary	18.86	15.99	3 to 40
Visual Perception	5.14	5.26	<1 to 14
Spatial Perception	2.90	6.41	<1 to 16
Visuoconstruction	3.07	3.86	<1 to 9
Verbal Learning/Memory			
Immediate Recall	28.71	22.52	<1 to 63
Delayed Recall	27.16	29.31	<1 to 75
Delayed Recognition	41.59	41.87	<1 to 84
Visual Learning/Memory			
Immediate Recall/Recognition	6.53	5.78	<1 to 16
Delayed Recall/Recognition	9.46	10.78	<1 to 37
Attention	5.62	7.58	<1 to 25
Working Memory	11.21	25.77	<1 to 84
Academic Skills			
Sight Word Reading	23.00	14.65	2 to 45
Math Computation	2.26	1.96	<1 to 5

*Note.* Percentile rank of 16^th^ and above is considered within normal limits or above, 3^rd^ to 15^th^ as below average, and ≤2^nd^ as low to very low.
