# Supplementary material for: Individuals with Wiedemann-Steiner syndrome show nonverbal reasoning and visuospatial defects with relative verbal skill sparing
Source: J Int Neuropsychol Soc. Author manuscript; Available in PMC 2025 Nov 26. (PMC12649766; doi:10.1017/S1355617722000467)
Supplement: Supplement [file NIHMS2119443-supplement-Supplement.doc]

Supplementary Table 1. Genetic information of our clinical sample with Wiedemann Steiner Syndrome.

| Patients (years in age at testing) | Inheritance | Variants | Mutation type | Molecular test completed | Pathogenicity stage |
| --- | --- | --- | --- | --- | --- |
| Patient 1 (6y) | De novo | c.4576-1G>C; IVS12-1G>C | Splice site | Trio WES 2018 | Pathogenic |
| Patient 2 (7y) | De novo | c.1260delG; p.I421Sfs*9 | Frameshift | Proband only WES 2016 | Pathogenic |
| Patient 3 (7y) | De novo | c.5873A>C p.H1958P | Missense | Trio WES 2019 | VUS |
| Patient 4 (9y) | De novo | c.7438C>T; p.R2480X | Nonsense | Trio WES 2017 | Pathogenic |
| Patient 5 (9y) | Unknown | c.11084C>G; p.S3695X | Nonsense | Single gene testing | Pathogenic |
| Patient 6 (11y) | Unknown | c.478C>T; p.R160X | Nonsense | Proband only WES 2017 | Pathogenic |
| Patient 7 (12y) | De novo | c.5251A>T; p.K1751X | Nonsense | Trio WES 2020 | Pathogenic |
| Patient 8 (13y) | De novo | c.1474_1493dup;  p. P500Lfs*74 | Frameshift | Trio WES 2016 | Pathogenic |
| Patient 9 (13y) | Inherited from 10% mosaic father | c.4013-2A>T; IVS7-2A>T | Splice site | Trio WES 2019 | Pathogenic |
| Patient 10 (17y) | De novo | c.3460C>T; p.R1154W | Missense | Trio WES 2015 | Pathogenic |
| Abbreviations: WES= whole exome sequencing. All variants are based on transcript NM_001197104.1. | | | | | |

| Patients (years in age) | Intel. Comp. (SS) | Verbal Index | Receptive  Vocabulary | Verbal Memory  Immediate | Verbal Memory  Delayed | Verbal Memory Recognition | Non-Verbal  Index | Visual Perception | Spatial Perception | Visuo-construction | Visual, Memory Immediate | Visual Memory, Delayed |
| --- | --- | --- | --- | --- | --- | --- | --- | --- | --- | --- | --- | --- |
| Patient 1 (6y*) | 88 | WNL | WNL | WNL | WNL | L | BA | BA | WNL | BA | BA | BA |
| Patient 2 (7y*) | --- | WNL | --- | --- | --- | --- | BA | --- | --- | L | BA | BA |
| Patient 3 (7y) | 69 | BA | WNL | WNL | BA | L | L | BA | L | BA | L | BA |
| Patient 4 (9y*) | --- | L | BA | WNL | WNL | WNL | L | L | --- | --- | BA | BA |
| Patient 5 (9y) | 67 | BA | --- | L | L | L | L | --- | --- | BA | WNL | BA |
| Patient 6 (11y) | 66 | L | BA | WNL | WNL | WNL | L | BA | L | L | BA | WNL |
| Patient 7 (12y*) | 81 | WNL | WNL | WNL | BA | WNL | BA | BA | L | L | L | BA |
| Patient 8 (13y*) | 64 | BA | WNL | WNL | BA | WNL | L | L | L | L | BA | BA |
| Patient 9 (13y*) | 52 | L | BA | WNL | BA | L | L | L | L | L | L | L |
| Patient 10 (17y) | 64 | BA | --- | L | WNL | WNL | L | L | --- | --- | --- | --- |
| Abbreviations**.**  Intel Comp = Intellectual Functioning Composite, SS=Standard Score, WNL=Within Normal Limits, BA= Below Average, L=Low to Very Low  Note. Overall intellectual functioning was not available for two patients. Patient 2 completed the DAS-II School-Age Verbal and Non-verbal cluster subtests through telehealth modality, but not Spatial measures as it requires manual manipulation of stimuli. Thus, an estimate of intellectual functioning was not possible. Patient 4 was administered the out-of-level core measures from the DAS-2 Early Years test battery, which does not yield an index of overall intellectual functioning. Within normal limits (WNL) refers to percentile rank ≥16. Below average (BA) refers to percentile rank 3 to 16. Low to very low (L) refers to percentile rank ≤ 2. Asterisk refers to patients with a diagnosis of intellectual disability. Asterisk (*) refers to those tested via telehealth modality. | | | | | | | | | | | | |

Supplementary Table 2. Percentile rank of patients with Wiedemann Steiner Syndrome across verbal and visual tests.
